# Cdk2 Is Required for p53-Independent G_2_/M Checkpoint Control

**DOI:** 10.1371/journal.pgen.1000863

**Published:** 2010-02-26

**Authors:** Jon H. Chung, Fred Bunz

**Affiliations:** Department of Radiation Oncology and Molecular Radiation Sciences and The Sidney Kimmel Comprehensive Cancer Center, Johns Hopkins University School of Medicine, Baltimore, Maryland, United States of America; Fred Hutchinson Cancer Research Center, United States of America

## Abstract

The activation of phase-specific cyclin-dependent kinases (Cdks) is associated with ordered cell cycle transitions. Among the mammalian Cdks, only Cdk1 is essential for somatic cell proliferation. Cdk1 can apparently substitute for Cdk2, Cdk4, and Cdk6, which are individually dispensable in mice. It is unclear if all functions of non-essential Cdks are fully redundant with Cdk1. Using a genetic approach, we show that Cdk2, the S-phase Cdk, uniquely controls the G_2_/M checkpoint that prevents cells with damaged DNA from initiating mitosis. *CDK2*-nullizygous human cells exposed to ionizing radiation failed to exclude Cdk1 from the nucleus and exhibited a marked defect in G_2_/M arrest that was unmasked by the disruption of *P53*. The DNA replication licensing protein Cdc6, which is normally stabilized by Cdk2, was physically associated with the checkpoint regulator ATR and was required for efficient ATR-Chk1-Cdc25A signaling. These findings demonstrate that Cdk2 maintains a balance of S-phase regulatory proteins and thereby coordinates subsequent p53-independent G_2_/M checkpoint activation.

## Author Summary

Metazoan cells contain multiple Cdks that regulate cell cycle progression. Recent studies have shown that mouse cells can grow normally with just Cdk1. The roles of the non-essential Cdks remain a fundamental question. In this study, we describe the generation and detailed characterization of *CDK2*-knockout human somatic cells. Our study demonstrates that Cdk2 is required for robust DNA damage checkpoint signaling. Loss of Cdk2 caused a marked deficiency in the G_2_/M arrest—a basic response to DNA damage—in cells that were also nullizygous for *P53*. We propose that the multiple Cdks present in metazoan cells provide a mechanism by which the cell cycle can be efficiently halted after DNA damage. Significantly, this study reveals a heretofore unrecognized dependence for Cdk2 in p53-deficient cancer cells.

## Introduction

Cdks associate with cyclins to form heterodimers that are sequentially activated during the cell cycle. Metazoan cells have multiple Cdks and cyclins that are temporally regulated [1],[2]. In normal cell cycles, Cdk4 and Cdk6 pair with D-type cyclins during G_1_, Cdk2 pairs with E- and A-type cyclins during S and G_2_, and Cdk1 pairs with A- and B-type cyclins during G_2_ and M. The importance of Cdks in cell cycle transitions was suggested by studies in which expression of dominant negative mutants or introduction of inhibitory antibodies or small molecule inhibitors caused phase-specific cell cycle arrest [3]. However recent genetic studies have called into question the requirement for multiple Cdks [3]–[5]. RNAi-mediated depletion of Cdks in human cells [6] and gene knockouts in mice [7]–[10] showed that Cdk2, Cdk4 and Cdk6 are dispensable for cell cycle progression. Cdk1 can bind D-, E-, and A-type cyclins and functionally substitute for the non-essential Cdks [11]. While it is clear that Cdk1 alone can drive unperturbed cell cycle progression, it remains unclear whether the non-essential Cdks have non-redundant functions in cell cycle responses to stress.

Cdks are targeted by checkpoints that halt the cell cycle in response to DNA damage. Cdk2 is primarily considered a downstream target of the S-phase checkpoint [12],[13]. However Cdk2 can also signal upstream via the phosphorylation of ATRIP, a binding partner of the ATR kinase [14]. Despite this data suggesting a role for Cdk2 in the regulation of checkpoint signaling, conflicting genetic evidence challenges the functional requirement for Cdk2 in DNA damage responses. Both the G_1_/S and G_2_/M checkpoints appear to remain fully functional in *CDK2^−/−^* mouse embryonic fibroblasts (MEFs) [15],[16]. As Cdk2 and Cdk1 are functionally redundant in supporting DNA replication, it would seem plausible that Cdk1 could similarly substitute for Cdk2 in checkpoint pathways. Such redundancy would account for the lack of apparent checkpoint defects in the mouse *CDK2-*knockout.

Here we show that Cdk2 uniquely activates the G_2_/M checkpoint and that this function is masked by the presence of p53, which functions independently to arrest cells in G_2_ after DNA damage. Unlike the functions of Cdk2 during unperturbed S-phase, the role of Cdk2 in the G_2_/M checkpoint is non-redundant and cannot be performed by Cdk1.

## Results

### Stabilization of Cdc25A in Cdk2-deficient cells

**Figure 1:**
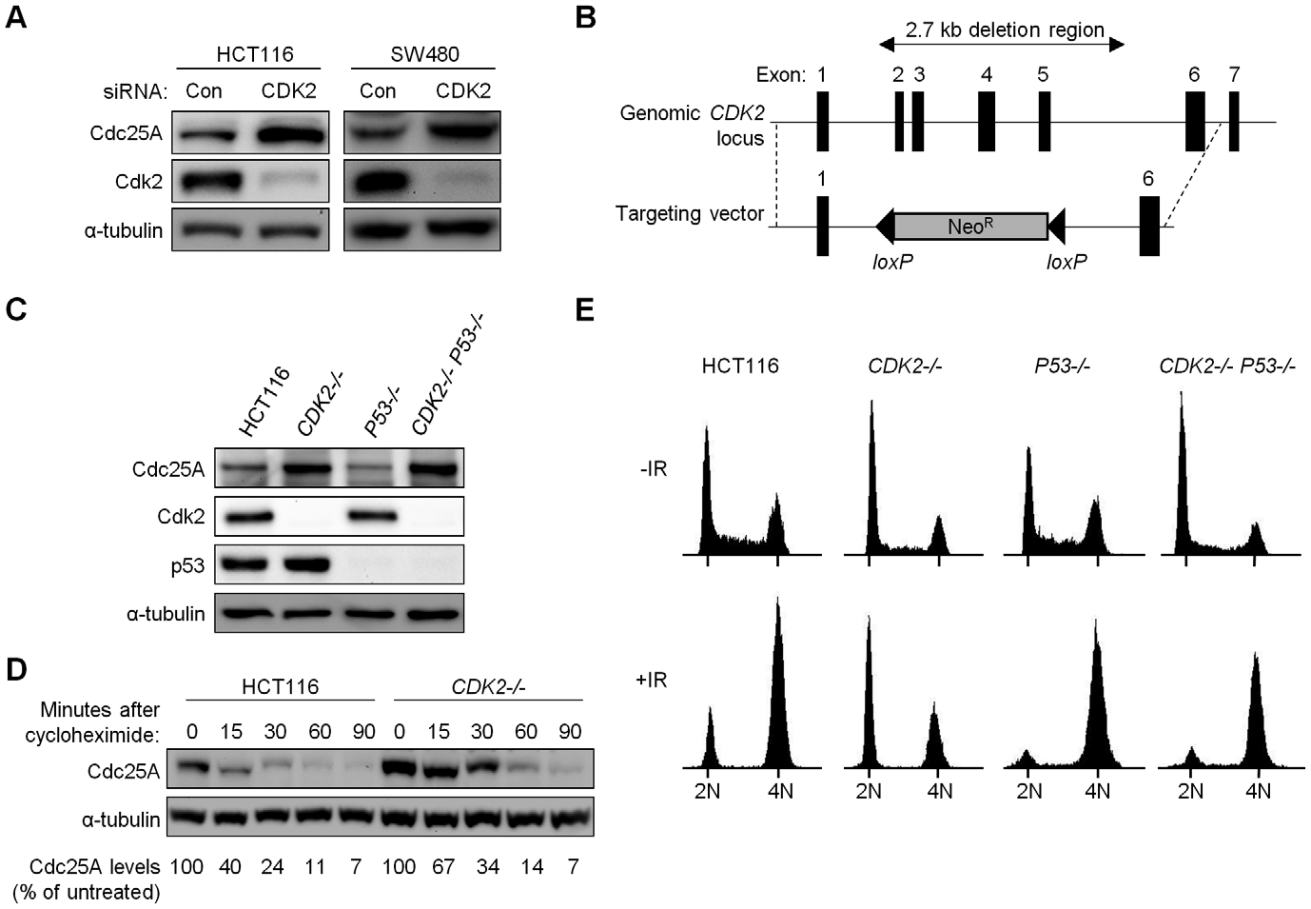
Altered cell cycle regulation and increased Cdc25A protein in Cdk2-deficient human cancer cells. (A) Human colorectal cancer cells HCT116 and SW480 transfected with control (Con) or *CDK2* (CDK2) siRNA. Levels of Cdc25A and Cdk2 were analyzed by immunoblot. α-tubulin was assessed as a loading control. (B) *CDK2* targeting strategy. Exons 2-5 at the *CDK2* locus were replaced upon integration of the knockout vector. (C) Levels of Cdc25A, Cdk2 and p53 were determined by immunoblot in parental cells and single and double knockout cells. (D) Isogenic HCT116 cells were harvested at the indicated times after cycloheximide treatment. Cdc25A protein levels were analyzed by immunoblot, quantitated, normalized to α-tubulin and represented as a percentage relative to untreated control cells. (E) Isogenic HCT116 cells were fixed and stained with Hoechst 33258 and subjected to flow cytometry, before and 24 h after treatment with 12 Gy IR. Positions of cell populations with 2N and 4N DNA content are indicated.

Does Cdk2 contribute to human checkpoints? We first tested whether Cdk2 is required for the regulation of Cdc25A, a common target of checkpoint kinases and a critical mediator of cell cycle transitions. Depletion of Cdk2 with siRNA resulted in increased Cdc25A protein levels in human colorectal cancer cells (Figure 1A).

To unambiguously evaluate the role of Cdk2 in checkpoint responses, we disrupted both *CDK2* alleles in the human colorectal cancer cell line HCT116 (Figure 1B). HCT116 cells have intact DNA damage-responsive checkpoints [17]–[19] and detailed analysis of these cells has revealed that p53 is required for maintaining stable arrest at G_1_/S and at G_2_/M after ionizing radiation (IR) [17]. To compare the contributions of p53 and Cdk2, we disrupted *P53* and *CDK2* individually to generate *P53^−/−^* and *CDK2^−/−^* cells, respectively, and together to generate double knockout cells (*CDK2^−/−^ P53^−/−^*). Two double knockout clones were obtained in independent experiments. As expected, homozygous disruption of *P53* and *CDK2* led to loss of protein expression in a genotype-specific manner (Figure 1C). Consistent with the established role of Cdk2 in promoting the G_1_-S transition, asynchronous *CDK2^−/−^* cells exhibited an elevated G_1_ fraction with fewer cells in S-phase (Figure 1E). Following IR treatment, 60% of *CDK2^−/−^* cells arrested at G_1_/S (Figure 1E), consistent with previous observations of an intact G_1_/S checkpoint in *CDK2^−/−^* MEFs [15],[16]. *P53* disruption caused a characteristic loss of the G_1_/S checkpoint, irrespective of *CDK2* genotype (Figure 1E). Stabilization of p53 and the induction of its downstream target p21 after IR were not affected by *CDK2* disruption (Figure S1C).

In *P53^+/+^* and *P53^−/−^* backgrounds, Cdk2 deficiency resulted in increased Cdc25A (Figure 1C). Cdc25A protein levels are known to be tightly controlled by phosphorylation, in both stressed and unstressed cells [20],[21]. To determine if increased Cdc25A protein following loss of Cdk2 was due to changes in stability, we assessed Cdc25A turnover by treating HCT116 and *CDK2^−/−^* cells with the protein synthesis inhibitor cycloheximide. While Cdc25A was degraded by 90 min in *CDK2^−/−^* cells, the rate of degradation was decreased (Figure 1D) indicating that Cdk2 contributes to normal Cdc25A protein turnover.

### Cdk2 and p53 cooperatively mediate G_2_/M checkpoint arrest

**Figure 2:**
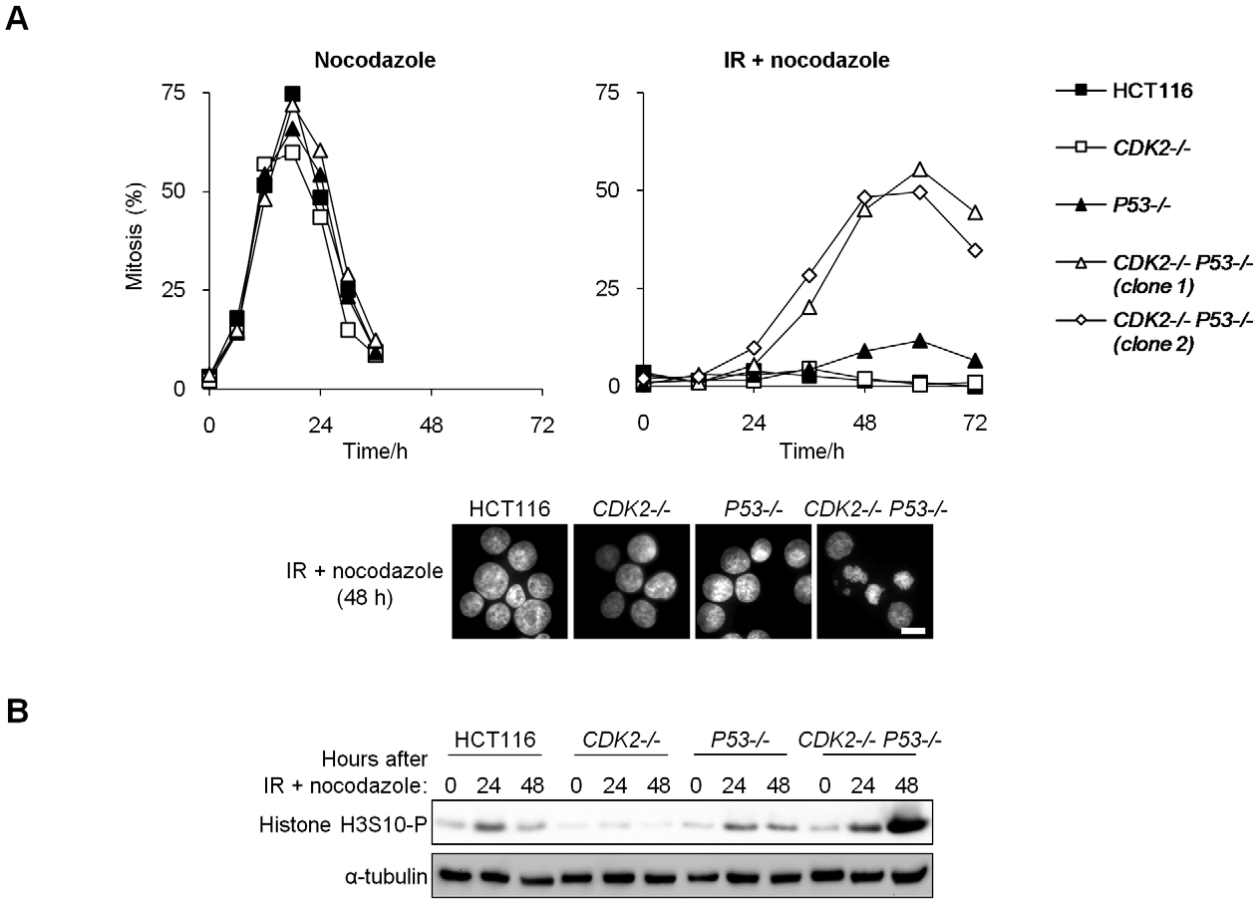
Cdk2 is required for G_2_/M arrest in p53-deficient cells. (A,B) Entry into mitosis was assessed by incubation of untreated or irradiated (12 Gy) isogenic HCT116 cells in 0.2 µg/ml nocodazole. Two independent *CDK2^−/−^ P53^−/−^* clones are shown. Cells were collected at the indicated times. (A) Cells were fixed and stained with Hoechst 33258. Mitotic chromosome condensation was determined by fluorescence microscopy, images were taken at 63× magnification (scale bar, 10 µm). (B) Levels of histone H3S10-P were determined by immunoblot.

To assess the integrity of the G_2_/M checkpoint response to DNA double strand breaks, we treated isogenic cultures with IR and trapped the cells that subsequently entered mitosis with the microtubule-destabilizing drug nocodazole. Cells of all genotypes arrested normally in mitosis when treated with nocodazole alone (Figure 2A). p53-deficient cells do not stably arrest at G_2_/M following IR [17], and therefore exhibited a modest increase in mitotic entry after 48–60 h, compared with wild type cells in which the mitotic index remained below 4% (Figure 2A). The extent of mitotic entry was greatly elevated in double knockout cells (*CDK2^−/−^ P53^−/−^*; Figure 2A). Accordingly, the mitotic marker phospho-histone H3 S10 (H3S10-P) was strongly expressed in *CDK2^−/−^ P53^−/−^* cells 48 h following IR/nocodazole treatment (Figure 2B). Unirradiated cells entered mitosis within 24 h of the addition of nocodazole (Figure 2A). The temporal delay in the mitotic entry of irradiated double knockout cells compared with unirradiated controls suggests that checkpoint pathways were activated in the absence of Cdk2 and p53, but were apparently insufficient to facilitate stable arrest. This G_2_/M checkpoint defect was apparent over a range of IR doses (Figure S1A) and could be detected as early as 24 h after IR/nocodazole treatment (Figure 2 and Figure S1A). In contrast, the majority of *CDK2* knockout-*P53* wild type cells (*CDK2^−/−^*) arrested at G_1_/S after IR treatment, and the remaining subpopulation (about 40%) of these cells arrested at G_2_/M with 4N DNA content (Figure 1E). A very small number of these 4N cells entered mitosis over the course of the experiment (Figure 2 and Figure S1A). We conclude that Cdk2 plays an important role in G_2_/M arrest after DNA damage and that the requirement for Cdk2 was masked by the function of p53 at both the G_1_/S and G_2_/M checkpoints.

### Cdk2-null cells fail to sequester Cdk1 in the cytoplasm after IR

The G_2_-M transition is controlled in part by Cdk1 localization. In unperturbed cells, Cdk1 is cytoplasmic during interphase and enters the nucleus in prophase to trigger mitosis. After DNA damage, Cdk1 is excluded from the nucleus, thus contributing to arrest in G_2_ [22],[23].

*CDK2* ^−/−^ MEFs have been reported to exhibit altered Cdk1 localization [15]. In these cells, deregulated Cdk1 localization has been attributed to the redistribution of cyclin E, normally associated with Cdk2, to Cdk1 [15]. Several studies demonstrate a similar redistribution of the nuclear protein cyclin A to Cdk1 in the absence of Cdk2 in both mouse and human cells [11],[15],[24],[25], and such complexes have been shown to promote mitotic entry [26]–[30].

**Figure 3:**
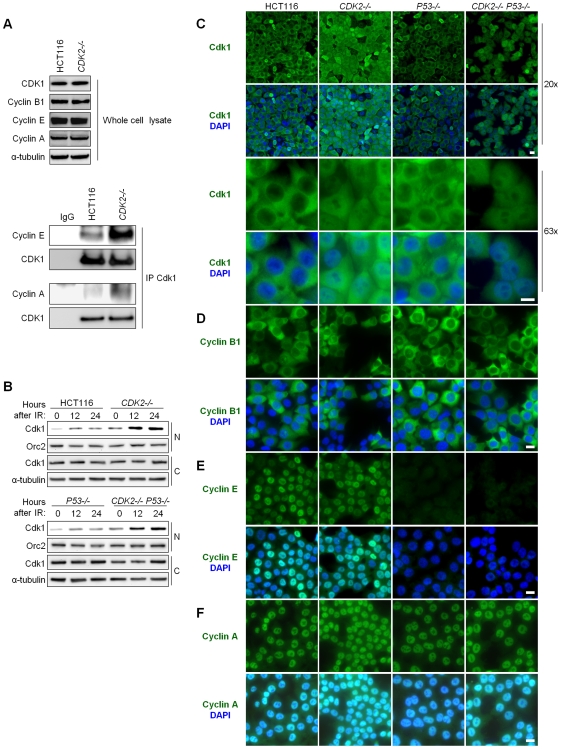
Aberrant localization of Cdk1 in Cdk2-deficient cells after IR treatment. (A) Association between Cdk1 and cyclins E and A was determined by immunoprecipitation/immunoblot. Non-denatured cell lysates were subjected to immunoprecipitation (IP) with anti-Cdk1 or control (IgG) antibodies. Samples not subjected to immunoprecipitation were analyzed as whole cell lysate. Levels of Cdk1 and cyclins B1, E, and A were determined by immunoblot. (B) Cdk1 levels in nuclear and cytoplasmic fractions were determined by immunoblot at the indicated times after treatment with 12 Gy IR. Loading of nuclear and cytoplasmic proteins was assessed by probing levels of Orc2 and α-tubulin, respectively. (C) Localization of Cdk1 by indirect immunofluorescence. Isogenic HCT116 cells were fixed 24 h after treatment with 12 Gy IR, stained with Cdk1 antibody (green) and counterstained with DAPI (blue). Representative fields are shown under low (20×) and high (63×) magnification (scale bar, 50 µm under 20× magnification and 10 µm under 63× magnification). (D–F) Localization of cyclins by indirect immunofluorescence. Isogenic HCT116 cells were fixed 24 h after treatment with 12 Gy IR, stained with (D) cyclin B1, (E) cyclin E or (F) cyclin A antibodies (green) and counterstained with DAPI (blue). Representative fields are shown under 40× magnification (scale bar, 10 µm).

Consistent with these previous studies, the amount of cyclin E and cyclin A associated with Cdk1 was increased in *CDK2*-knockout human cells (Figure 3A). As Cdk localization is dependent on its partner cyclin [31],[32], we asked whether the changes in Cdk1-cyclin complexes observed in *CDK2^−/−^* cells might affect Cdk1 localization.

Total Cdk1 protein levels were unaffected by *CDK2* genotype or IR (Figure 3A and Figure S1C). After IR treatment, the amount of Cdk1 in the nucleus was increased in *CDK2* ^−/−^ cells compared to wild type cells (Figure 3B). The increase in nuclear Cdk1 was independent of *P53* genotype, and temporally preceded entry of double knockout cells into mitosis (Figure 3B). Together, these data suggest that aberrant nuclear Cdk1 was a cause rather than a consequence of defective G_2_/M checkpoint function in *CDK2^−/−^ P53^−/−^* cells. The failure of *CDK2-*knockout cells to exclude Cdk1 from the nucleus in response to IR was confirmed by immunofluorescence. The localization of Cdk1 in untreated cells was similar in HCT116 and all isogenic derivatives (data not shown). By 24 h after IR, virtually all cells with wild type *CDK2* had sequestered Cdk1 in the cytoplasm, while *CDK2^−/−^* cells exhibited Cdk1 staining in both the nuclear and cytoplasmic compartments (Figure 3C).

To determine which cyclin partners might contribute to the altered Cdk1 localization in *CDK2^−/−^* cells, we examined the localization of cyclin B1, cyclin A and cyclin E after IR. Cyclin B1 was cytoplasmic in all cell lines (Figure 3D), suggesting that aberrant nuclear localization of Cdk1 was not caused by deregulated cyclin B1 localization. In contrast, cyclins E and A were nuclear in checkpoint-proficient *P53*-wild type cells (Figure 3E and 3F). Cyclin E was barely detectable in *P53^−/−^* cells after IR (Figure 3E), presumably because these cells bypass the G_1_/S checkpoint and progress to G_2_/M wherein cyclin E is not normally expressed; cyclin A, which is normally expressed from interphase until prometaphase, was located in the nucleus in these cells (Figure 3F). In agreement with studies of *CDK2^−/−^* MEFs [15], these findings suggest that the redistribution of cyclin E and cyclin A to Cdk1 results in its aberrant localization to the nucleus after DNA damage.

### Deregulation of Cdc25A is a cause of G_2_/M checkpoint failure

In addition to localization, Cdk1 is also controlled by inhibitory phosphorylation. The Cdc25A phosphatase promotes mitotic entry by removing the inhibitory Y15 phosphate moiety on Cdk1 (Cdk1Y15-P) [33]. This mode of activation is turned off following IR, when Cdc25A is degraded in a Chk1-dependent manner [20]. Cdk1 Y15 phosphorylation is a p53-independent checkpoint mechanism [33].

**Figure 4:**
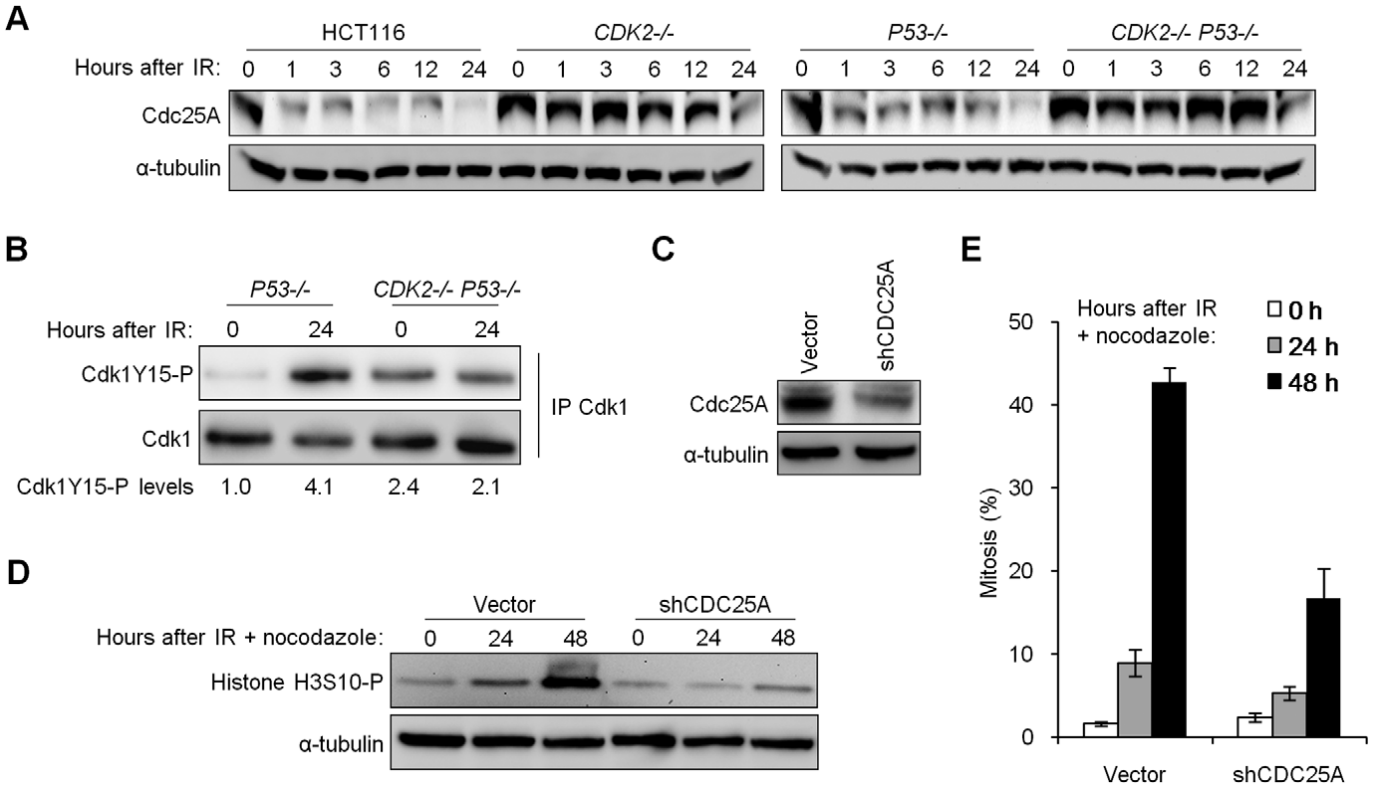
Impaired degradation of Cdc25A in Cdk2-deficient cells contributes to G_2_/M checkpoint defect. (A) Isogenic HCT116 cells were harvested at the indicated times after treatment with 12 Gy IR. Cdc25A levels were determined by immunoblot. (B) Non-denatured cell lysates were collected before and 24 h after 12 Gy IR. Cdk1 was immunoprecipitated (IP) and analyzed for phosphorylation on Y15 by immunoblot. Cdk1Y15-P was quantitated and normalized to total Cdk1. (C) Stable knockdown of Cdc25A in *CDK2^−/−^ P53^−/−^* cells by retrovirus-mediated delivery of empty vector control (vector) or *CDC25A* shRNA (shCDC25A), assessed by immunoblot. (D) Cdc25A knockdown or control *CDK2^−/−^ P53^−/−^* cells were treated with 12 Gy IR and 0.2 µg/ml nocodazole. Histone H3S10-P levels were determined by immunoblot. (E) Mitotic indices of Cdc25A-knockdown and control *CDK2^−/−^ P53^−/−^* cells at the indicated times after treatment with 12 Gy IR and 0.2 µg/ml nocodazole. Error bars represent s.e.m. from six knockdown clones.

The increased Cdc25A protein levels in untreated, Cdk2-deficient cells (Figure 1C and 1D) prompted us to examine whether Cdc25A was also aberrantly regulated in response to IR. While Cdc25A was rapidly degraded after IR in wild type cells, Cdc25A levels remained high in cells lacking Cdk2 (Figure 4A). Next, we asked whether the failure to degrade Cdc25A after IR affected the ability of *CDK2*-knockout cells to induce Cdk1Y15-P and arrest at G_2_/M. As expected, p53-deficient cells normally induced Cdk1Y15-P after IR (Figure 4B); in contrast, the levels of Cdk1Y15-P did not increase after IR in cells that were also Cdk2-deficient. Interestingly, the phosphorylation of Cdk1 Y15 was increased in untreated Cdk2-deficient cells compared to untreated cells with wild type Cdk2 (Figure 4B). This increase in basal Cdk1Y15-P could be a consequence of the redistribution of Cdk1 to alternative Cdk1-cyclin heterodimers and the consequent expansion of the role of Cdk1 to multiple phases of the cell cycle, in Cdk2-deficient cells (Figure 3A) [11],[15],[24],[25]. It is unknown if non-canonical Cdk1-cyclin heterodimers are efficient substrates for activating phosphatases and inhibitory kinases.

To assess the relevance of increased Cdc25A protein levels to the observed checkpoint defect, we tested whether stable depletion of Cdc25A could restore checkpoint function. Depletion of Cdc25A in *CDK2^−/−^ P53^−/−^* cells using short hairpin RNAs (Figure 4C) suppressed histone H3S10-P induction (Figure 4D) and mitotic chromosome condensation (Figure 4E) after IR/nocodazole treatment. Mitotic entry of unirradiated cells was not affected by Cdc25A knockdown (Figure S1B). These results show that elevated Cdc25A contributes significantly to the checkpoint defect.

### Cdk2 facilitates ATR-Chk1-Cdc25A pathway activation in part by stabilizing Cdc6

Cdc25A protein stability is regulated by two pathways in response to IR. ATM phosphorylates the checkpoint kinase Chk2 [34], which then triggers Cdc25A degradation [12]. Cdc25A is also targeted for IR-dependent degradation by Chk1 [21], which is activated after phosphorylation by ATR on residues S317 and S345 [35],[36]. The ATR-Chk1 signaling pathway is active at a reduced physiological level during unperturbed cell growth, and regulates basal Cdc25A protein turnover during S-phase [21].

**Figure 5:**
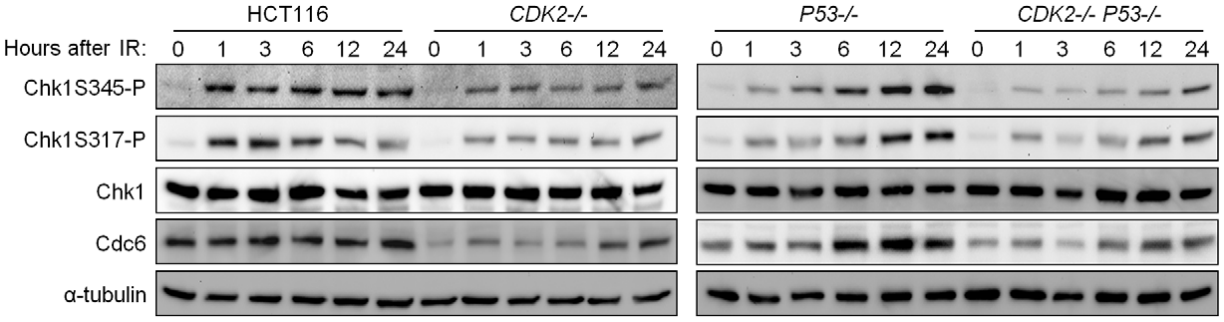
Impaired phosphorylation of Chk1 in Cdk2-deficient cells. Isogenic cells were treated with 12 Gy IR and harvested at the indicated time points. Levels of the Chk1 phosphoproteins, Chk1S345-P and Chk1S317-P, Chk1 and Cdc6 were determined by immunoblot. Levels of α-tubulin were assessed as a loading control.

IR-induced phosphorylation of Chk1 S317 and S345 was reduced in *CDK2*-knockout cells, irrespective of *P53* genotype (Figure 5). Levels of IR-induced Chk1 S345 and S317 phosphorylation (Chk1S345-P and Chk1S317-P) were stably high in wild type cells but increased over time in p53-deficient cells. This effect is likely due to the entry of G_1_/S checkpoint-defective *P53^−/−^* cells into S-phase (Figure 1C), when ATR activity is known to increase [37]. RNAi-mediated knockdown of Cdk2 in a diverse panel of human cell lines consistently reduced IR-dependent Chk1 phosphorylation (Figure S2A). In contrast, Chk2 phosphorylation by ATM and the formation of DNA damage foci containing phosphorylated ATM were unaffected by *CDK2* genotype (Figure S1C and S1D). These observations suggest that Cdk2 is required for efficient ATR- but not ATM-mediated checkpoint signaling.

To determine how Cdk2 might control ATR signaling, we first examined the status of ATR localization and known interacting proteins. The ability of ATR to localize to DNA damage foci was unaffected by disruption of *CDK2* (Figure S2C), as was the interaction between ATR and ATRIP, the requisite ATR binding partner [38] (Figure S2D). ATRIP is phosphorylated on residue S224 by Cdk2 [14] and this phosphorylation event has been shown to contribute to G2/M checkpoint arrest. We observed that cells lacking Cdk2 exhibited somewhat reduced ATRIP S224 phosphorylation (Figure S2B). Given the modest deficiency in ATRIP phosphorylation, we investigated additional mediators that might also contribute to checkpoint signaling by Cdk2.

One compelling candidate is the Cdk2 substrate Cdc6. Cdc6 is a loading factor for the DNA replicative helicase complex required for replication origin licensing [39]. In addition to its role in DNA replication, Cdc6 has also been implicated as a regulator of checkpoint function and mitotic entry [39]–[44]. In human cells, depletion of Cdc6 causes cells with actively replicating DNA to aberrantly enter mitosis [42], while overexpression of Cdc6 causes Chk1 phosphorylation and G_2_/M arrest [40]. Cdc6 has also been implicated in ATR-Chk1 signaling in fission yeast [41]and *Xenopus* [44].

**Figure 6:**
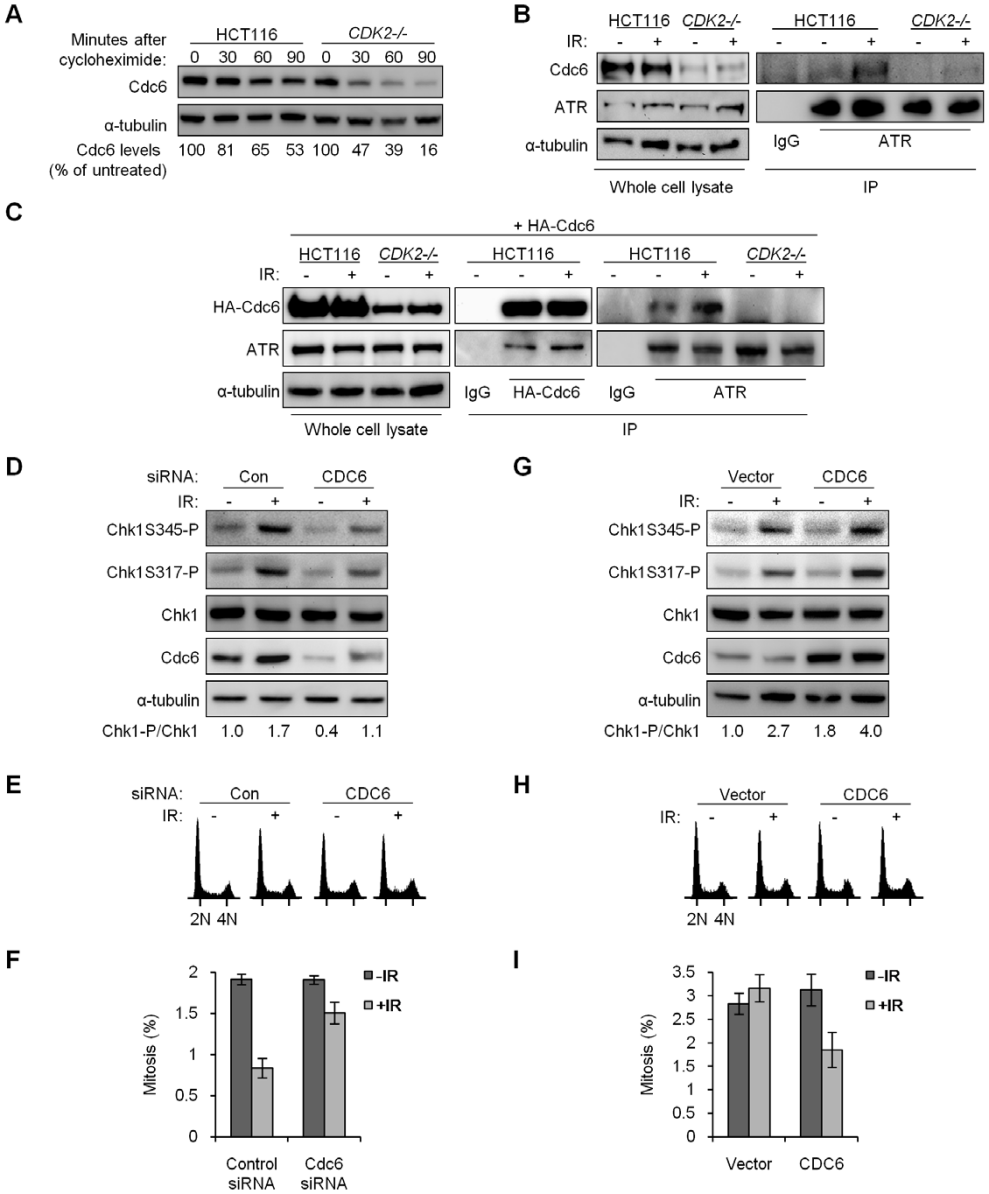
Stabilized Cdc6 facilitates Chk1 phosphorylation, associates with ATR, and controls mitotic entry. (A) Cdc6 protein stability was analyzed by immunoblot in wild-type and *CDK2^−/−^* cells at the indicated times after cycloheximide treatment. Cdc6 levels were quantitated, normalized to α-tubulin, and represented as a percentage relative to untreated control cells. (B) Physical interaction between endogenous Cdc6 and ATR. Non-denatured cell lysates from the indicated cell lines were collected before or 4 h after 12 Gy IR. Lysates were subjected to immunoprecipitation (IP) with anti–ATR or isotypic control (IgG) antibodies. Samples not subjected to immunoprecipitation were analyzed as whole cell lysate. Levels of ATR and Cdc6 were determined by immunoblot. (C) Isogenic HCT116 cells were transfected with a plasmid expressing HA-tagged Cdc6 (HA-Cdc6). Transfected cells were untreated or treated with 12 Gy IR and lysed after 4 h. Non-denatured cell lysates were subjected to immunoprecipitation (IP) with anti–HA, anti–ATR, or isotypic control (IgG) antibodies. Samples not subjected to immunoprecipitation were analyzed as whole cell lysate. Levels of ATR and HA-Cdc6 were determined by immunoblot. (D–F) U2OS cells were transfected with control (Con) or *CDC6* (CDC6) siRNA. (D) Lysates were taken before and 4 h after treatment with 12 Gy IR. Chk1 and Cdc6 levels were analyzed by immunoblot. Relative Chk1-phosphorylation (Chk1-P/Chk1) was determined by quantitation of Chk1S317-P plus Chk1S345-P followed by normalization to total Chk1. (E) Cells were fixed and stained with Hoechst 33258 and subjected to flow cytometry before and 4 h after treatment with 12 Gy IR. (F) Cells fixed and stained for histone H3S10-P before and 24 h after treatment with 4 Gy IR and 1 µg/ml nocodazole, mitotic index was determined by indirect immunofluorescence. Error bars represent s.e.m. from three independent experiments. (G–I) *CDK2^−/−^ P53^−/−^* cells were transfected with an empty vector or Cdc6 expression vector. (G) Lysates were taken before and 4 h after treatment with 12 Gy IR. Chk1 and Cdc6 levels were analyzed by immunoblot. (H) Cells were fixed and stained with Hoechst 33258 and subjected to flow cytometry before and 4 h after treatment with 12 Gy IR. (I) Cells fixed and stained with Hoechst 33258 before and 24 h after treatment with 12 Gy IR and 0.2 µg/ml nocodazole, mitotic index was determined by fluorescence microscopy. Error bars represent s.e.m. from six independent experiments.

Inherently unstable, Cdc6 can be stabilized as a direct result of phosphorylation by Cdk2 [45]. Although Cdc6 is an essential DNA replication protein, cells lacking functional Cdk2 are able to progress through S-phase despite significantly reduced Cdc6 levels [6],[45]. Therefore the relatively small amount of Cdc6 remaining in Cdk2-deficient cells is clearly sufficient to support DNA replication and cell cycle progression. In concordance with previous studies, Cdc6 protein levels were decreased (Figure 5 and Figure S3A), and turnover was increased (Figure 6A), in Cdk2-deficient cells. In checkpoint-deficient *P53^−/−^* cells, Cdc6 levels increased after IR in tandem with Chk1 phosphoprotein (Figure 5). These results are consistent with a potential role for Cdc6 in the regulation of the upstream kinase of Chk1, ATR.

In fission yeast, a direct interaction between the Cdc6 homologue Cdc18 and the ATR homologue Rad3 is induced in response to replication stress; this complex then activates checkpoint signaling [41]. ATR and Cdc6 also interact following replication stress in human cells [43]. We asked whether Cdc6 and ATR might similarly interact after IR. Complexes of endogenous ATR and Cdc6 were detected after IR in HCT116 cells (Figure 6B). To confirm this interaction we exogenously expressed HA-tagged Cdc6 (HA-Cdc6; [45]) in HCT116 and *CDK2*-knockout cells and probed for an interaction with ATR (Figure 6C). Exogenous expression of HA-Cdc6 was higher in wild type cells (Figure 6C), which mirrored the relative abundance of endogenous Cdc6 and further illustrated the stabilizing effect of Cdk2 (Figure 6B and Figure S3A). ATR co-precipitated with HA-Cdc6, and the amount of ATR bound increased after IR treatment (Figure 6C, Figure S3C and S3D). In the reciprocal experiment, ATR was able to pull down increased HA-Cdc6 after IR treatment in both HCT116 and U2OS cell lines (Figure 6C and Figure S3B). The coimmunoprecipitation of Cdc6 and ATR was not disrupted by 50 µg/ml ethidium bromide, suggesting that this interaction is specific and not simply mediated by DNA (data not shown).

We could not reliably detect ATR-Cdc6 complexes in *CDK2* ^−/−^ cells, before or after IR (Figure 6B and 6C). As Cdc6 stability and protein levels were markedly decreased in *CDK2*-knockout cells (Figure 6A–6C and Figure S3A), we asked whether the lack of detectable interaction was simply due to reduced Cdc6 protein levels or, alternatively, if loss of Cdk2-dependent phosphorylation on Cdc6 directly disrupted its interaction with ATR. Mutant Cdc6 proteins, wherein the Cdk2-phosphorylated serine residues (S54, S74, S106) were replaced either with non-phosphorylatable alanine residues (HA-Cdc6AAA) or with phosphomimetic aspartic acid residues (HA-Cdc6DDD; [45]), were expressed and pulled down. ATR co-precipitated with HA-Cdc6AAA in wild type HCT116 cells and with HA-Cdc6DDD in *CDK2^−/−^* cells (Figure S3C and S3D). These results indicate that the ATR-Cdc6 interaction is independent of Cdc6 phosphorylation by Cdk2 per se, and that the differences in complex formation observed were most likely the result of decreased Cdc6 levels caused by Cdk2 deficiency.

To examine whether Cdk2-mediated stabilization of Cdc6 could functionally contribute to ATR-Chk1 signaling, we experimentally manipulated Cdc6 levels. First, we knocked down Cdc6 by siRNA. Cdc6 protein levels could be transiently lowered in U2OS cells, which express wild type p53, by siRNA transfection (Figure 6D).

The effects of Cdc6 levels on cell growth have been intensively studied. Depending on the extent and timing of Cdc6 depletion and the type of target cell, Cdc6 knockdown has been shown to result in variable changes to cell cycle distribution as well as cell death [42], [46]–[51]. In many cases, transient depletion of Cdc6 in various cell types, including HCT116 [46],[48],[51], has been reported to have minimal effects. Normal cells and cancer cells have been observed to respond differently to Cdc6 knockdown [49],[52], but the genetic alterations in cancer cells that might underlie such differences have not been conclusively identified. We observed that changes to the cell cycle distribution 48 h after partial Cdc6 knockdown were minimal, with a similar proportion of cells in S-phase and a small decrease of cells in G_1_ (Figure 6E). Knockdown of Cdc6 caused a reduction in IR-induced Chk1 phosphorylation (Figure 6D) that was reminiscent of the observed changes in checkpoint signaling after *CDK2* knockdown (Figure S2A) or knockout (Figure 5). While knockdown of Cdc6 was less efficient in HCT116 cells, decreased Cdc6 also led to reduced IR-induced Chk1 phosphorylation, irrespective of *P53* genotype (Figure S3E). Knockdown of Cdc6 also led to increased levels of the Chk1 target Cdc25A, before and after IR treatment (Figure S3F and S3G). To determine if Cdc6 protein levels could functionally impact G_2_/M checkpoint arrest, we assessed mitotic index after *CDC6* knockdown followed by sequential treatment with IR and nocodazole. Twenty-four hours after IR/nocodazole treatment, cells pretreated with *CDC6* siRNA entered mitosis in higher numbers as compared with control siRNA (Figure 6F).

We next increased Cdc6 levels by transient transfection of an untagged Cdc6 expression construct. While partial restoration of Cdc6 expression in *CDK2^−/−^ P53^−/−^* knockout cells did not appreciably alter the cell cycle profile (Figure 6H), it did result in increased IR-induced Chk1 phosphorylation (Figure 6G) and reduced mitotic entry after IR/nocodazole treatment (Figure 6I). Together, the results of these overexpression and knockdown experiments suggest that stabilization of Cdc6 by Cdk2 contributes to efficient IR-induced Chk1 phosphorylation by ATR and p53-independent G_2_/M checkpoint function. The Cdk2-Cdc6 pathway appears to have a direct affect on ATR-Chk1 signaling, as cell cycle profiles were only minimally changed by Cdc6 manipulation under these conditions (Figure 6E and 6H).

## Discussion

**Figure 7:**
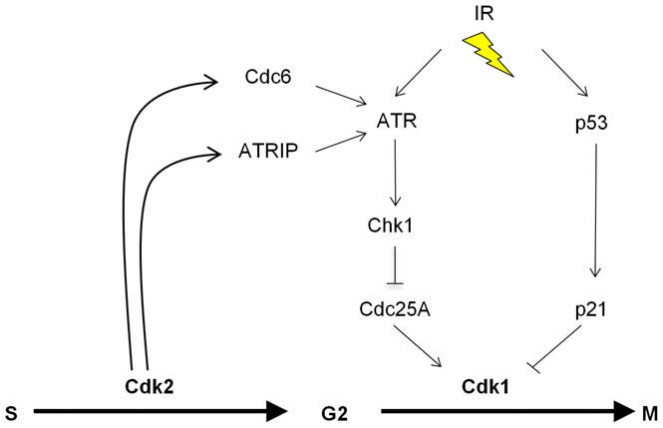
The proposed role of Cdk2 in the p53-independent regulation of Cdk1. Cdk2 directly phosphorylates at least two proteins, ATRIP and Cdc6, that directly regulate the G_2_-M transition via the ATR-Chk1-Cdc25A pathway. The activity of Cdk1 is independently inhibited upon activation of the p53-p21 pathway, which is defective in many cancer cells. See text for additional details.

Studies of knockout mice have now unequivocally shown that the essential S-phase functions previously attributed to Cdk2 can also be conducted by Cdk1 in somatic cells [3]–[5]. These seminal observations raised the question of why mammalian cells express multiple Cdks that appear to be non-essential. In this report, we demonstrate that loss of Cdk2 alters the regulation of several proteins that are known to regulate S-phase progression, but also control mitotic entry, including Cdc25A, Chk1, Cdc6 and ATRIP [33],[39],[53]. The altered balance of these bifunctional proteins did not affect the transition of *CDK2^−/−^* cells through the phases of the unperturbed cell cycle, but did compromise their ability to mount effective checkpoint signaling through the ATR-Chk1 pathway. Cdk2 and Cdk1 are therefore redundant with respect to essential cell cycle functions, but have distinct, non-redundant roles in a key DNA damage response. IR-induced ATR activation is restricted to the S- and G_2_-phases [37], when Cdk2 is normally active. We show that Cdk2 plays a unique role in facilitating robust DNA damage checkpoint control by the ATR-Chk1-Cdc25A pathway. Cdk2 appears to promote the formation of active ATR complexes in at least two ways: via the phosphorylation of ATRIP and by the stabilization of Cdc6 (Figure 7). It is possible that Cdk2 also controls checkpoint signaling through additional mechanisms such as the recently described Cdk2 interacting protein (CINP) which facilitates robust ATR signaling [54].

At present the precise mechanism by which Cdc6 affects ATR signaling remains unclear. Our data suggest that Cdk2-mediated phosphorylation of Cdc6 merely regulates Cdc6 levels but is not otherwise required for ATR-Cdc6 complex formation (Figure S3C). In agreement with our results, a paper published during the preparation of this manuscript has reported an interaction between ATR and Cdc6 in human and *Xenopus* cells [51] and showed that the non-phosphorylatable Cdc6-AAA mutant could interact with ATR, albeit with somewhat lower efficiency. The fission yeast Cdc6 homologue Cdc18 is required to anchor the ATR homologue Rad3 to chromatin [41], but our results suggest that Cdc6 may not perform an analogous function in human cells (Figure S2B). Further study is required to determine if Cdc6 might directly affect ATR catalytic activity, or if Cdc6 might promote the assembly of higher order complexes required for full ATR activation.

In conjunction with checkpoint pathways that target Cdk1 via Cdc25 phosphatases, the exclusion of Cdk1 from the nucleus is an important G_2_/M checkpoint mechanism [23]. In mouse [15] and human (Figure 3B and 3C) cells lacking Cdk2, Cdk1 becomes aberrantly localized to the nucleus. It would therefore appear that the formation of non-canonical Cdk1-cyclin heterodimers that allow Cdk1 to compensate for Cdk2 in the completion of S phase in unperturbed cells [11],[24],[25] also impairs the ability of damaged cells to arrest at G_2_/M. We propose that the temporal division of respective S-phase and G_2_-phase functions between Cdk2 and Cdk1 is a critical feature of the metazoan cell cycle that allows its progress to be efficiently halted after DNA damage.

Defective checkpoints are a feature of the majority of human cancers [55]–[57]. In many cancers, checkpoint deficiencies are caused by loss-of-function mutations in *P53*. The genetic interaction between *P53* and *CDK2* described here demonstrates a novel, non-redundant requirement for Cdk2 in the p53-independent G_2_/M checkpoint pathways that remain intact in cancer cells.

## Materials and Methods

### Gene targeting

Endogenous *CDK2* and *P53* loci were disrupted in HCT116 cells using recombinant adeno-associated virus (rAAV)-based gene targeting methods [58],[59]. Briefly, the targeting constructs pAAV-CDK2 and pSEPT-p53 [58] were packaged into infectious rAAV subsequently used to generate transgenic clones. Identification and expansion of homologous recombinant cell lines was performed as described [59]. At least two independent clones were isolated and analyzed for each cell line.

### Cell culture, siRNA, and cell cycle analysis

HCT116, SW480 and derivatives were cultured in McCoys 5A supplemented with 6% FCS. U2OS cells were cultured in DMEM supplemented with 10% FCS. *CDK2* and *CDC6* siRNA pools and non-targeting control pools were purchased from Dharmacon. Transfections were performed with 100 nM siRNA and Lipofectamine 2000 (Invitrogen). In HCT116, optimal Cdk2 knockdown was achieved by two transfections 48 h apart and cells were analyzed 96 h after initial transfection. In SW480, optimal Cdk2 knockdown was achieved by a single transfection and cells were analyzed after 72 h. Cdc6 knockdown was achieved by a single transfection and cells were analyzed after 48 h. IR and nocodazole treatment were performed as described [17]. For cell cycle analysis cells were fixed, stained with Hoechst 33258 and analyzed by flow cytometry or microscopy for mitotic chromosome condensation as described [17]. Mitotic index for U2OS cells were determined by immunofluorescence for histone H3S10-P staining.

### Protein analysis

Total cell lysates were prepared using NuPAGE sample buffer (Invitrogen). Non-denatured cell lysates for immunoprecipitation were collected in Cell Lysis Buffer (Cell Signaling). Immunoprecipitations were performed by incubation of lysates with antibody and Protein A/G PLUS-Agarose beads (Santa Cruz) overnight at 4°C. Beads were washed, resuspended and boiled in NuPAGE lysis buffer. Proteins were separated on NuPAGE gels (Invitrogen), transferred to PVDF membranes, probed with antibodies and developed using Enhanced Chemiluminescence (Amersham). Primary antibodies were directed against α-tubulin, ATR, Cdc6, Cdk1, Cdk2, Chk1, cyclin A, cyclin E, cyclin B1, HA, p53 (Santa Cruz), Cdc25A (Neomarkers), ATMS1981-P, Cdk1Y15-P, Chk1S317-P, Chk1S345-P, Chk2T68-P (Cell Signaling), ATRIP, histone H3S10-P (Millipore), Orc2 (BD Biosciences), and ATRIPS224-P (a gift from D Cortez), as indicated. The Quantity One 4.6.1 software package (Bio-Rad) was used for quantitation. For analysis of protein stability, cells were incubated in 100 µg/ml cycloheximide prior to lysis; band intensities were measured and normalized to α-tubulin abundance. Protein levels were expressed as a percentage of untreated control cells.

### Cell fractionation and immunofluorescence microscopy

Subcellular fractionation was performed as described [60]. For immunofluorescence, cells were grown on chamber slides and fixed with 3.75% paraformaldehyde/2% sucrose. Fixed cells were permeabilized in 0.2–0.5% Triton X-100 and blocked in BSA. Immunofluorescence staining was performed using Cdk1, cyclin A, cyclin B1, cyclin E or histone H3S10-P antibodies followed by biotin-conjugated secondary antibody (Santa Cruz) and Alexa-488 conjugated avidin (Molecular Probes). Cells were counterstained with 4′-6′-diamidino-2-phenylindole (DAPI) and mounted with Fluoromount-G (Southern Biotech). Images were captured at room temperature using an AxioImager Z1 microscope equipped with an AxioCam HRm camera, Axiovision 4.6.3 software, and a Plan Neofluar 20x/0.25NA, 40x/1.3NA or 63x/1.25NA lens (Zeiss), as indicated. Images were processed for brightness and contrast using Adobe Photoshop.

### Retroviral gene transfer

*CDC25A* shRNA was cloned from pSUPER-Cdc25A [61] into the retroviral plasmid pBabe to generate pBabe-shCDC25A. Retroviral production using Amphopack293 cells (Clontech) and subsequent gene transfer was performed according to protocols supplied by the manufacturer.

### Plasmids

Plasmids encoding HA-Cdc6, HA-Cdc6AAA and HA-Cdc6DDD were previously described [45]. To generate the untagged full length Cdc6 construct, human Cdc6 cDNA was cloned into pCDNA3.1/Hygro (Invitrogen). Cells were transfected using Lipofectamine 2000 (Invitrogen) and analyzed after 24–48 h.

## Supporting Information

Figure S1 Checkpoint signaling in HCT116 cells and isogenic derivatives in response to ionizing radiation. (A) Isogenic HCT116 cells were treated with 5 Gy, 8 Gy, or 12 Gy IR followed by 0.2 µg/ml nocodazole. Cells were fixed and stained with Hoechst 33258. Mitotic index was assessed by counting condensed chromosomes. Error bars represent s.e.m. from three independent experiments. (B) *CDK2^−/−^ P53^−/−^* clones stably expressing *Cdc25A* shRNA from Figure 4E were treated with 0.2 µg/ml nocodazole. Mitotic index was assessed at the indicated times. Error bars represent s.e.m. from six clones. (C) Isogenic cells were harvested after treatment with 12 Gy IR. Levels of Chk2T68-P, Cdk1, p53, p21 and α-tubulin were determined by immunoblot. (D) HCT116 and *CDK2^−/−^* cells were fixed with 3.75% paraformaldehyde and permeabilized with 0.5% Triton-X-100. Fixed cells were stained using anti-ATMS1981-P antibody and counterstained with DAPI. Cells were fixed before and 3 h after treatment with 10 Gy IR. Images were captured at 63× magnification (scale bar, 10 µm).
(1.01 MB TIF)

Figure S2 Effects of Cdk2 deficiency on the ATR-Chk1 pathway. (A) Levels of the indicated proteins were determined by immunoblot in human U2OS osteosarcoma cells, MCF7 breast adenocarcinoma cells, H1299 lung adenocarcinoma cells, and SW480 colorectal adenocarcinoma cells before and after treatment with 12 Gy IR. U2OS and MCF7 lysates were collected 0.5 h after IR. H1299 and SW480 lysates were collected 2 h after IR. Cells transfected with siRNA directed against *CDK2* (CDK2) were compared with cells transfected with control siRNAs (Con). (B) Levels of ATRIPS224-P in untreated isogenic HCT116 cells were determined by immunoblot, quantitated and normalized to α-tubulin. (C) HCT116 and *CDK2^−/−^* cells were simultaneously fixed and permeabilized with 4% paraformaldehyde and 0.5% Triton-X-100. Fixed cells were stained using anti–ATR antibody and counterstained with DAPI. Cells were fixed before and 20 h after treatment with 12 Gy IR. Images were captured at 63× magnification (scale bar, 10 µm). (D) Non-denatured cell lysates from the indicated cell lines were collected before or 4 h after 12 Gy IR. Lysates were subjected to immunoprecipitation (IP) with anti-ATR or control (IgG) antibodies. Samples not subjected to immunoprecipitation were analyzed as whole cell lysate. Levels of ATR and ATRIP were determined by immunoblot.
(0.58 MB TIF)

Figure S3 Interaction of Cdc6 with ATR and modulation of Chk1-Cdc25A signaling. (A) Levels of Cdc6 were determined by immunoblot in the indicated isogenic cell lines. (B–D) Non-denatured cell lysates were collected before or 4 h after treatment with 12 Gy IR. Lysates were subjected to immunoprecipitation (IP) with anti–ATR, anti–HA, or IgG isotype control antibodies. Lysates not subjected to immunoprecipitation were analyzed as Whole Cell Lysate. Levels of ATR and HA-Cdc6 in were determined by immunoblot. Lysates for immunoprecipitation were collected from (B) U2OS cells transfected with a plasmid expressing HA-Cdc6, (C) HCT116 transfected with a plasmid expressing HA-Cdc6 or HA-tagged non-phosphorylatable Cdc6 (HA-Cdc6-AAA), and (D) HCT116 and *CDK2^−/−^* transfected with a plasmid expressing HA-Cdc6 or HA-tagged phosphomimetic Cdc6 (HA-Cdc6-DDD). (E) Chk1 levels were determined by immunoblot in isogenic HCT116 cells transfected with control (Con) or CDC6 (CDC6) siRNA. Lysates were taken before and 4 h after treatment with 12 Gy IR. (F) Cdc25A levels were determined by immunoblot in U2OS or isogenic HCT116 cells transfected with control or CDC6 siRNA. (G) Cdc25A levels were determined by immunoblot in U2OS cells transfected with control or CDC6 siRNA before and 4 h after treatment with 12 Gy IR.
(0.47 MB TIF)
