# Supplementary figures and images for: Cdk2 Is Required for p53-Independent G2/M Checkpoint Control
Source: PLoS Genet. 2010 Feb 26;6(2):e1000863. doi: 10.1371/journal.pgen.1000863 (PMC2829054; doi:10.1371/journal.pgen.1000863)

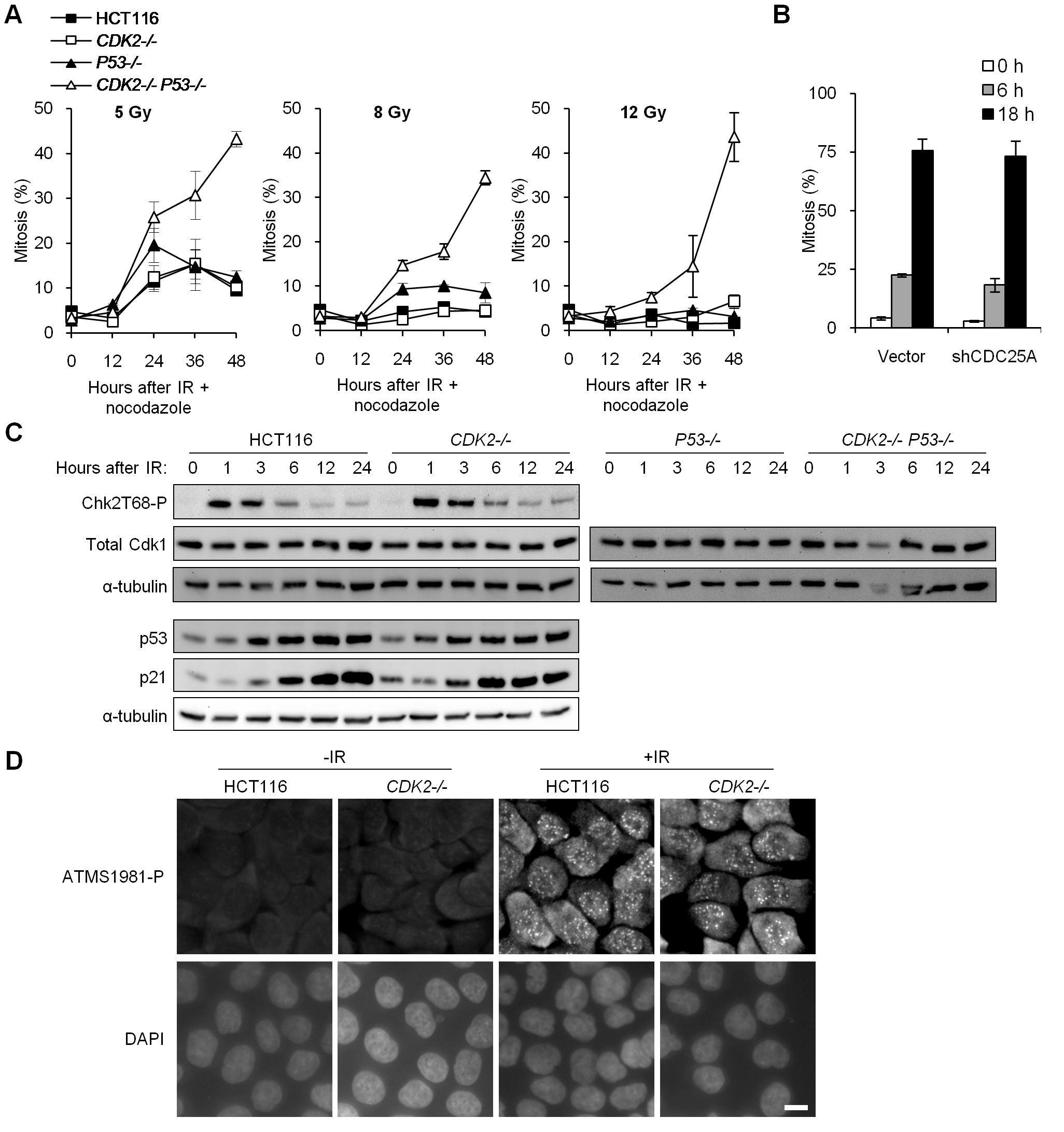

Supplement: Figure S1 — Checkpoint signaling in HCT116 cells and isogenic derivatives in response to ionizing radiation. (A) Isogenic HCT116 cells were treated with 5 Gy, 8 Gy, or 12 Gy IR followed by 0.2 µg/ml nocodazole. Cells were fixed and stained with Hoechst 33258. Mitotic index was assessed by counting condensed chromosomes. Error bars represent s.e.m. from three independent experiments. (B) CDK2−/− P53−/− clones stably expressing Cdc25A shRNA from Figure 4E were treated with 0.2 µg/ml nocodazole. Mitotic index was assessed at the indicated times. Error bars represent s.e.m. from six clones. (C) Isogenic cells were harvested after treatment with 12 Gy IR. Levels of Chk2T68-P, Cdk1, p53, p21 and α-tubulin were determined by immunoblot. (D) HCT116 and CDK2−/− cells were fixed with 3.75% paraformaldehyde and permeabilized with 0.5% Triton-X-100. Fixed cells were stained using anti-ATMS1981-P antibody and counterstained with DAPI. Cells were fixed before and 3 h after treatment with 10 Gy IR. Images were captured at 63× magnification (scale bar, 10 µm). (1.01 MB TIF) [file pgen.1000863.s001.tif]

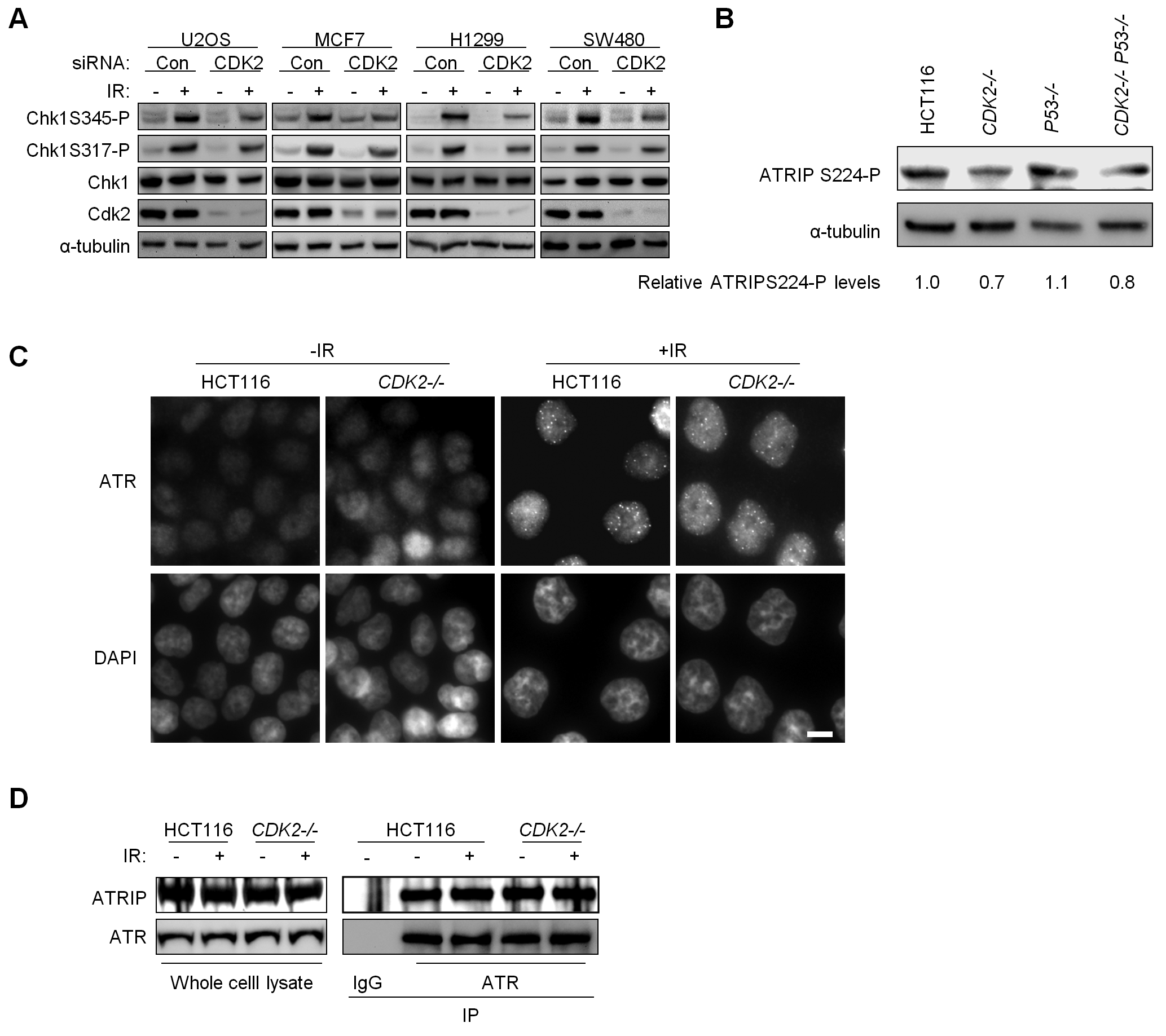

Supplement: Figure S2 — Effects of Cdk2 deficiency on the ATR-Chk1 pathway. (A) Levels of the indicated proteins were determined by immunoblot in human U2OS osteosarcoma cells, MCF7 breast adenocarcinoma cells, H1299 lung adenocarcinoma cells, and SW480 colorectal adenocarcinoma cells before and after treatment with 12 Gy IR. U2OS and MCF7 lysates were collected 0.5 h after IR. H1299 and SW480 lysates were collected 2 h after IR. Cells transfected with siRNA directed against CDK2 (CDK2) were compared with cells transfected with control siRNAs (Con). (B) Levels of ATRIPS224-P in untreated isogenic HCT116 cells were determined by immunoblot, quantitated and normalized to α-tubulin. (C) HCT116 and CDK2−/− cells were simultaneously fixed and permeabilized with 4% paraformaldehyde and 0.5% Triton-X-100. Fixed cells were stained using anti–ATR antibody and counterstained with DAPI. Cells were fixed before and 20 h after treatment with 12 Gy IR. Images were captured at 63× magnification (scale bar, 10 µm). (D) Non-denatured cell lysates from the indicated cell lines were collected before or 4 h after 12 Gy IR. Lysates were subjected to immunoprecipitation (IP) with anti-ATR or control (IgG) antibodies. Samples not subjected to immunoprecipitation were analyzed as whole cell lysate. Levels of ATR and ATRIP were determined by immunoblot. (0.58 MB TIF) [file pgen.1000863.s002.tif]

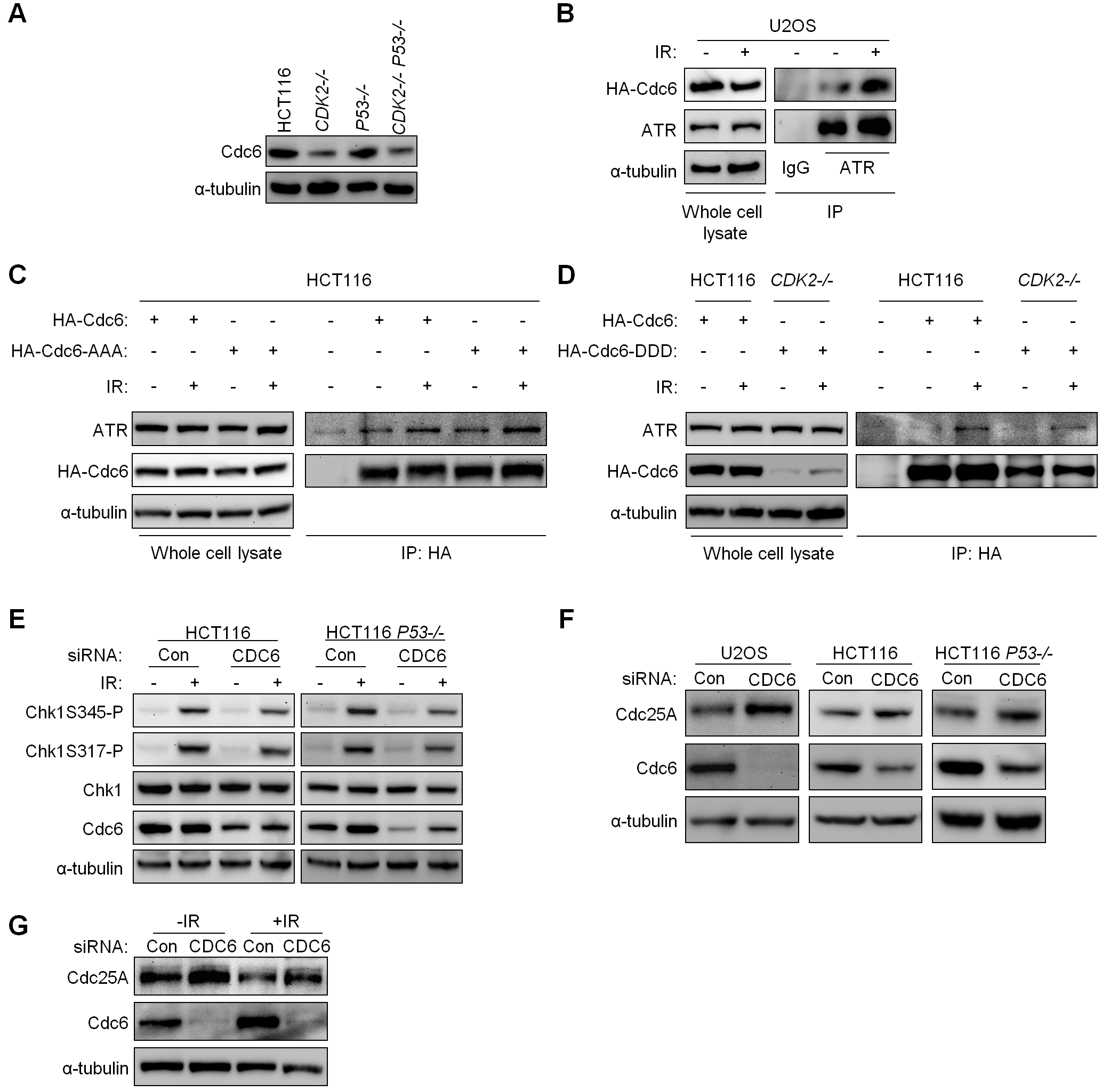

Supplement: Figure S3 — Interaction of Cdc6 with ATR and modulation of Chk1-Cdc25A signaling. (A) Levels of Cdc6 were determined by immunoblot in the indicated isogenic cell lines. (B–D) Non-denatured cell lysates were collected before or 4 h after treatment with 12 Gy IR. Lysates were subjected to immunoprecipitation (IP) with anti–ATR, anti–HA, or IgG isotype control antibodies. Lysates not subjected to immunoprecipitation were analyzed as Whole Cell Lysate. Levels of ATR and HA-Cdc6 in were determined by immunoblot. Lysates for immunoprecipitation were collected from (B) U2OS cells transfected with a plasmid expressing HA-Cdc6, (C) HCT116 transfected with a plasmid expressing HA-Cdc6 or HA-tagged non-phosphorylatable Cdc6 (HA-Cdc6-AAA), and (D) HCT116 and CDK2−/− transfected with a plasmid expressing HA-Cdc6 or HA-tagged phosphomimetic Cdc6 (HA-Cdc6-DDD). (E) Chk1 levels were determined by immunoblot in isogenic HCT116 cells transfected with control (Con) or CDC6 (CDC6) siRNA. Lysates were taken before and 4 h after treatment with 12 Gy IR. (F) Cdc25A levels were determined by immunoblot in U2OS or isogenic HCT116 cells transfected with control or CDC6 siRNA. (G) Cdc25A levels were determined by immunoblot in U2OS cells transfected with control or CDC6 siRNA before and 4 h after treatment with 12 Gy IR. (0.47 MB TIF) [file pgen.1000863.s003.tif]
